# Departure, routing and landing decisions of long-distance migratory songbirds in relation to weather

**DOI:** 10.1098/rsos.221420

**Published:** 2023-02-08

**Authors:** Georg Rüppel, Ommo Hüppop, Sander Lagerveld, Heiko Schmaljohann, Vera Brust

**Affiliations:** ^1^ Institute of Avian Research ‘Vogelwarte Helgoland’, 26386 Wilhelmshaven, Germany; ^2^ Wageningen Marine Research, 1781 AG Den Helder, The Netherlands; ^3^ Institute for Biology and Environmental Sciences, Carl von Ossietzky University Oldenburg, 26129 Oldenburg, Germany

**Keywords:** bird migration, stopover, departure decision, routing, landing decision, weather

## Abstract

Migrating birds flexibly adjust their individual migratory decisions, i.e. departing, routing and landing, based on intrinsic (e.g. energy stores) and extrinsic (e.g. landscape features and weather) factors modulating the endogenous stimuli. So far, these decisions have mostly been studied separately. Notably, we lack information on which factors landing decisions during active flight are based on. Therefore, we simultaneously recorded all three decisions in free-flying long-distance migratory songbirds in a coastal stopover area via regional-scale radio-telemetry and related them to the prevailing weather. Birds departed under favourable weather conditions resulting in specific nights with increased departure probability. Once departed, birds could either fly offshore or take a route along the coast, which was predicted by wind support. Radio-tracking revealed that departed individuals more likely interrupted their migratory endurance flight under overcast or headwind conditions. Studying departure, routing and landing decisions in concert, we highlight the importance of weather as a common driver across all migratory decisions. By radio-tracking individuals between stopovers, we provide evidence that avoidance of adverse weather conditions is an important function of stopover. Understanding how birds adjust migratory decisions and how they affect the timing of migration and survival is key to link migration performance to individual fitness.

## 1. Introduction

Each year, billions of birds migrate between their European breeding sites and African wintering grounds, interconnecting the two continents via the Western Palaearctic-African landbird flyway [1,2]. Most of them alternate between times on the ground, so-called stopovers, and flight bouts [3]. At stopover, migratory birds decide (i) when to resume migration and (ii) which route to take. During migratory endurance flight, they decide (iii) when to land and may adjust routing [4]. Since the time birds spend during stopovers is generally far longer than the total duration of migratory endurance flights, frequency and duration of stopovers largely determine the overall speed and progress of migration [5–8]. Departure decisions, selected flight routes and landing decisions, thus, crucially affect arrival timing at the destination [9–11]. Migration timing in turn affects survival and reproductive fitness based on migration strategy, season, sex and age [3,12,13]. Consequently, birds are assumed to flexibly adjust their migratory decisions to optimize overall migration performance [14].

Many migratory songbird species migrate mainly or even exclusively during the night (e.g. [15,16]) and primarily use daytime for resting, recovering or refuelling (e.g. [3,17]). Daily departure and landing decisions, hence, inextricably define the potential time spent flying during a migratory flight bout and, in combination with routing decision, the corresponding distances covered [3,18]. Therefore, studying all three decisions in concert is crucial to better understand individual variation in migration strategies. In songbirds, the overall spatio-temporal migration pattern is endogenously controlled [19,20], while individuals react flexibly to extrinsic conditions, e.g. atmospheric conditions, along the route [21] and adjust their migratory behaviour according to intrinsic factors, e.g. energy stores [22–24] and immune function [25,26]. For example, weather conditions that induce high metabolic costs of migratory endurance flights, such as headwinds, could lead to lower survival [27]. Thus, birds should wait for conditions that are more favourable for migration and adjust stopover timing accordingly.

Moreover, migratory decisions are not limited to stopover timing, but also include the routing decision. Routing not only affects the overall migration distance, it is again directly linked to survival due to potential threats along different routes [28,29] and spatial migration patterns are highly flexible among and within individuals [30,31]. Individual routing decisions become especially important in the context of barrier crossings, e.g. when landbirds negotiate crossing large water bodies. Since migratory songbirds are incapable of resting on water, they have to fly non-stop over water until they reach land, are vulnerable to risks resulting from deteriorating weather conditions and early exhaustion [29,32,33], and therefore should respond carefully to the current weather conditions for their migratory decisions.

Although weather conditions, primarily wind and precipitation, are known to affect migration patterns [34–37] and individual departure decisions [24,38–40], there still is only limited information about what actually initiates stopovers, i.e. the landing decision, especially in night-migratory songbirds due to methodological issues [3]. Nevertheless, understanding the proximate reasons for landing decisions is crucial to identify the actual functions of stopover. To fill parts of this gap in knowledge, we jointly investigated departure, routing and landing decisions of three migratory songbird species, Garden Warbler *Sylvia borin*, Greater Whitethroat *Curruca communis* and Sedge Warbler *Acrocephalus schoenobaenus*, at a coastal stopover site in Central Europe during autumn migration. These species are long-distance migrants wintering in Africa south of the Sahara and have similar migration distances [41].

**Figure 1.**
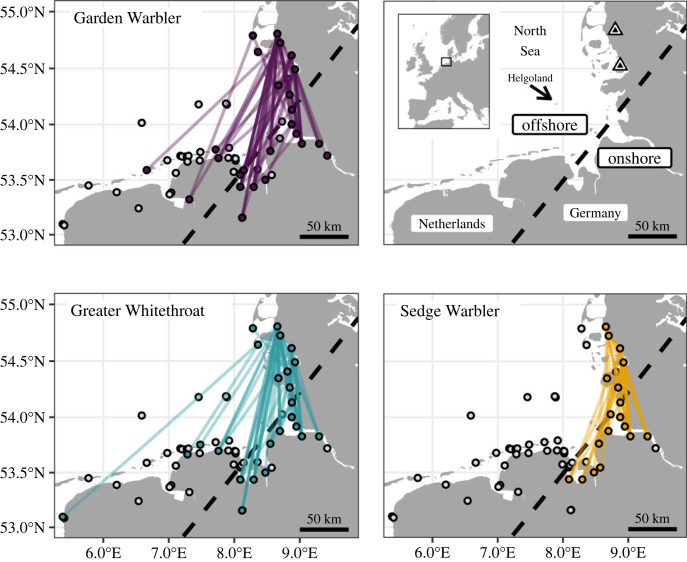
Maps of the study area illustrating individually detected flights per species (shown are begin and end locations of each flight connected by straight lines). Circles indicate receiver locations of the radio-telemetry network within the map section operational during the study (for temporal changes, see www.motus.org). The locations of tag deployment are indicated by triangles. Dashed black lines indicate threshold latitude and longitude for flight categorization as offshore (to the west) or onshore (to the east). Location of the island of Helgoland is indicated.

All birds were individually tracked by a regional-scale automated radio-telemetry network [42] covering more than 300 km of the southeastern coast of the North Sea (figure 1). The scale of the receiver network enabled us to track birds between stopovers and thus to study individual migratory decisions, including the landing decision, in unprecedented detail. Specifically, we were able to (i) detect individual departures from an initial stopover site, (ii) differentiate between offshore flights, i.e. sea-crossings, and onshore flights, i.e. flights along the coastline, and (iii) identify landings within the study area after a sustained flight. We were particularly interested in the underlying mechanisms for decisions regarding migration timing and routing. Focusing on extrinsic factors, we therefore estimated the relationships between all three migratory decisions and prevailing weather conditions. Birds were expected to minimize costs of transport by avoiding adverse environmental conditions for migratory endurance flights. Hence, departure decisions should be connected to favourable migratory conditions, e.g. wind support and no rain [32,43], routing should be adjusted according to wind support [36], and deteriorating conditions during migratory endurance flights, e.g. headwinds, may trigger landing decisions.

## 2. Methods

### 2.1. Study sites, study species and radio-tracking

We caught 275 songbirds (93 Garden Warblers, 95 Greater Whitethroats and 87 Sedge Warblers) with mist nets at the Beltringharder Koog (54.53°N, 8.88°E) and Lake Gotteskoog (54.84°N, 8.78°E) in Schleswig-Holstein, Germany (figure 1). Catching periods ranged from 16 to 31 August during the years 2019–2021 and thus lay well within the local autumn migration season of the three species [44]. Directly after catching, birds were fitted with uniquely coded radio-transmitters of type NTQB2-1 (burst intervals ranging between 4.5 and 8 s, Lotek Wireless Inc., Newmarket, ON, Canada) using leg-loop harnesses adjusted to body size [45]. Radio-transmitters including harness weighed 0.32 g and did not exceed 3.4% of the lowest birds' body mass recorded in this study. Since body masses could only be measured at tag deployment and most individuals departed on subsequent days, we do not consider influence of fuel stores, since they are unknown for most departures. Birds were immediately released at the catching site and tracked by a network of automated radio receiving stations covering much of the coastline and most islands along the German Bight and reaching into The Netherlands and Belgium (figure 1). The range over which tags can be detected by the receivers is estimated to be between 5 and 15 km [46], but varies considerably with the height and angle of the transmitting antenna to the receiving antenna, landscape and several weather parameters, e.g. temperature. All receiver stations are part of the global collaborative Motus Wildlife Tracking System [42]; for more information, see www.motus.org.

Detection data were processed by and retrieved from Motus using the *motus* R-package [47] and presumably false positives were discarded following the filtering routine described in Brust *et al*. [39]. We identified continuous movements (hereafter referred to as ‘flights’) within the individual tracking data. Following Brust *et al*. [39], a movement was defined as flight when it covered a distance of at least 35 km or was recorded by a minimum of three different receivers with consecutive detections in less than 7 h (information on the number of detections, and receivers with detections per individual flight are presented in electronic supplementary material, S1). In most cases, birds were passing by all including the last receiver station, indicating that they were still in flight, and the data represent only portions of longer flight bouts. For 18 birds, more than one flight was detected, but only the first flight of a bird was included in subsequent analyses to avoid pseudoreplication. Although we received signals from all tags at the stopover site, we did not record flights in 97 birds, which might be due to loss of tag, end of battery lifetime, predation or birds moving inland, and thus leaving the detection range. Every flight was categorized as either offshore or onshore (figure 1). An offshore oriented flight was defined to begin at geographical latitudes north of 54.135°N and to end at geographical longitudes west of 8.08°E, with no detections along the coastline between these two points (figure 1), or included detections on the island of Helgoland, which is located at least 50 km off the mainland [39]. We calculated the birds' minimum stopover duration as the time difference between initial capture, i.e. tag deployment, and the detected beginning of the flight. Although actual arrival times were unknown, it is reasonable to assume that most birds were probably caught briefly after arrival at the catching sites, because (i) catching took place on a daily basis throughout the catching periods, (ii) based on our field impression, catching success was aligned to the prevailing weather conditions in the previous night, i.e. migrants were more likely to be captured after clear nights with supporting winds than during windy, cloudy and rainy conditions, and (iii) estimated stopover durations already were quite long as compared to other studies (electronic supplementary material, S3) [48]. A landing was assumed if the bird interrupted its flight within the network of receiver stations (figure 1). We defined this by (i) detections at a certain receiver station for more than one hour or (ii) slow movements (less than 5 ms^−1^ [49]) with consecutive detections within 3 days at ranges of less than 32 km [50]. By choosing these thresholds, we excluded detections elsewhere in the further course of migration while we assumed that all birds with no detections within this range resumed migration and therefore had left the study area.

### 2.2. Weather data

Hourly weather data were obtained from ERA5 reanalysis accessed via the Copernicus Climate Change Service in a 0.25° × 0.25° horizontal grid [51,52] including eastward (u) and northward (v) wind component, temperature, atmospheric pressure and total cloud cover. Since songbird migration in Central Europe is concentrated to low elevations, i.e. 0–1500 m above sea level [53] and birds depart from the ground, all parameter values applied to near-surface level. Hourly precipitation data (binary index rain versus no rain) from 18 different weather stations were accessed via the Open Data Server of the German Weather Service [54,55]. All parameters were extracted individually for the location and time where each flight began and ended by considering the corresponding data of the respective grid cell or nearest weather station (maximum distance less than 20 km). To model daily departure decisions, we fetched the above weather parameters for each bird at the locations from where its flights began for each sunset (rounded to the full hour) until its departure. Additionally, we calculated the changes in both wind components, temperature and atmospheric pressure within the last 24 h.

### 2.3. Statistical analysis

#### 2.3.1. Departure

To identify factors which influence the individual and daily departure decisions from an initial stopover, we performed a proportional hazards model with M-splines baseline hazard [56] using the function stan_surv of the R-package *rstanarm* [57]. Time steps were defined as single days until departure. All weather parameters were included for individual locations from where the flights began at sunset for each day. The quadratic effects of both wind components were added to improve model fit using orthogonal polynomials. We added random intercepts for all year–species combinations and the day of year referring to tag deployment to formally correct for non-independent sampling. Since sampling took place within a rather short time period each year we here do not aim to show influence of season on migratory behaviour.

#### 2.3.2. Routing

As we were interested in the conditions that influence birds to decide whether to migrate offshore or fly along the coastline (figure 1), we modelled the binary decision (offshore versus onshore flight) using a logistic regression. All weather parameters at a bird's flight start location and departure time were used as explanatory variables. Additionally, we included random intercepts for all year–species combinations and the day of year referring to date of departure to formally correct for non-independent sampling. Sedge Warblers were not included in this model since we did not detect any offshore flights in this species.

#### 2.3.3. Landing

To assess the relationship between landing probability and certain weather parameters, we implemented a logistic regression with the binary landing decision as response variable. All weather parameters at a bird's flight end location and estimated landing time together with the minimal stopover duration prior to the flight were used as explanatory variables. The changes in both wind components between begin and end of each flight were used as additional covariates. All year–species combinations and the day of year, referring to the date of estimated flight end, were included as random intercepts to formally correct for non-independent sampling.

All data analyses were performed using R v.4.1.2 [58]. To obtain the joined posterior distributions of linear models, we used the function brm of the package *brms* keeping the default prior distributions [59]. For more details on model formulation and sampling, see the electronic supplementary material. All continuous explanatory variables were centred and scaled to one s.d. before analysis. To account for species-specific effects, we included two-way interactions between weather parameters and species in the first two models if they improved model fit substantially. Due to the low number of detected landings, we did not include interaction terms in the third model. Model fit and model assumptions were assessed via visual posterior predictive checks and model selection was based on leave-one-out cross-validation using the package *loo* [60]. The model assumptions were not violated. We report posterior distribution means and 95% credible intervals. Posterior probabilities are given as the proportion of simulated values from the posterior distribution that is larger than zero.

## 3. Results

### 3.1. Departure

**Figure 2.**
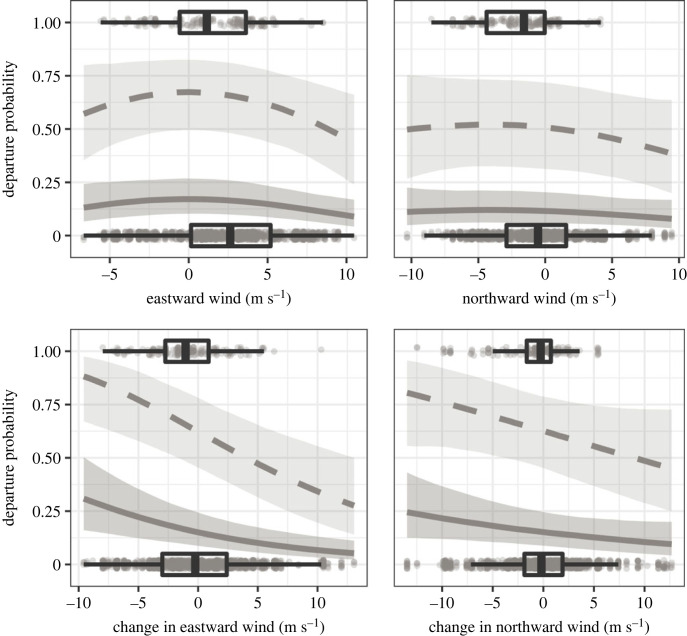
Time-dependent influence of wind on the departure decision from stopover during autumn, predicted by the proportional hazards model. Predictions are given for the 25% (solid lines) and 75% quantiles (dashed lines) of observed minimum stopover duration of 6 and 12 days together with 95% credible intervals (shaded areas). For time-dependent results in more detail, see electronic supplementary material, S6. Predictions were made for rainless conditions with the remaining numeric model covariates set to their means. Estimates are given in table 2. Raw data of observed parameter values per daily departure decisions of each bird from tag deployment until departure are indicated by dots and boxplots.

**Figure 3.**
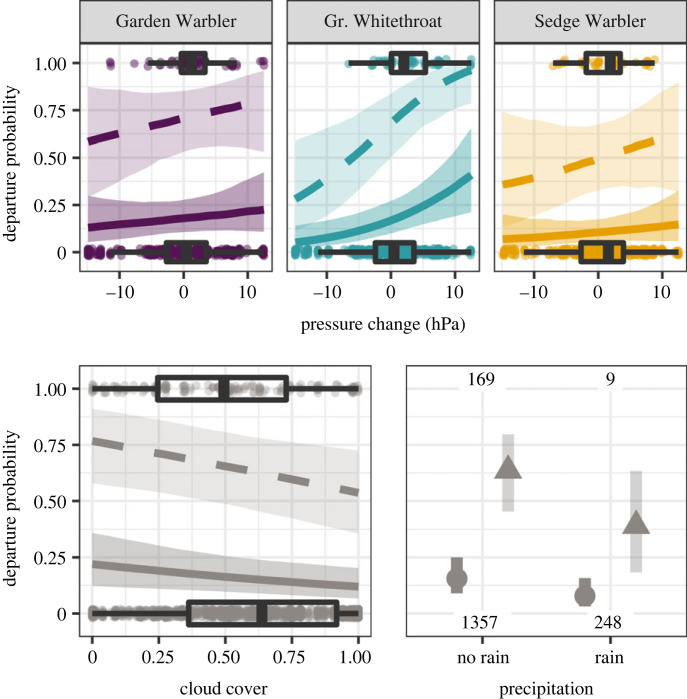
Time-dependent influence of weather parameters on the departure decision from stopover during autumn, predicted by the proportional hazards model. Predictions are given for the 25% (solid lines or dots) and 75% quantiles (dashed lines or triangles) of observed minimum stopover duration of 6 and 12 days together with 95% credible intervals (shaded areas, bars). For time-dependent results in more detail, see electronic supplementary material, S6. Predictions for pressure change and cloud cover were made for rainless conditions, and all remaining numeric model covariates were set to their means. Estimates are given in table 2. Raw data of observed parameter values per daily departure decisions of each bird from tag deployment until departure are indicated by dots and boxplots or given as the number of observed cases.

In total, we analysed 178 flights within our study area (figure 1, table 1), taking into account only the first flight per individual. Seven Garden Warblers and one Greater Whitethroat departed within the night after tag deployment, the median of the minimum stopover duration was 9 days (25% quantile: 6 days, 75% quantile: 12 days) with maxima of 22 days in one Sedge Warbler (electronic supplementary material, S3). There were six nights in which at least 10 birds departed, representing 40% of all detected flights. The most parsimonious model included both wind components and their changes, change in atmospheric pressure, cloud cover, precipitation and species as explanatory variables (table 2). Night-to-night departure probability peaked at light westward and southward winds, and increased with negative changes in the eastward wind component (i.e. a drop in eastward winds or increasing westward winds) and the northward wind components (i.e. a drop in northward winds or increasing southward winds) (figure 2). Departure probability further increased with positive changes in air pressure (varying between species), less cloud cover and absence of precipitation (figure 3). With the exception of air pressure change, responses among species were similar. Time series of weather parameters and departure activity are given in the electronic supplementary material, S4–S6.

**Table 1.** Number of detected sustained flights per year, together with routing and landing decisions of individually tracked night-migratory songbirds given per species.

species	2019	2020	2021	onshore	offshore	landings
Garden Warbler	18	25	23	51	15	5
Greater Whitethroat	17	26	25	59	9	12
Sedge Warbler	13	17	14	44	0	7

**Table 2.** Influence of weather parameters, species and interactions on night-to-night departure decision in Garden Warblers, Greater Whitethroats and Sedge Warblers. Estimates, 95% credible intervals and posterior probabilities for β > 0 are given for the final proportional hazards survival model.

parameter	estimate	2.5%	97.5%	*P*(β > 0)
intercept	1.73	1.21	2.26	1.00
eastward wind (linear)	−3.48	−7.70	0.77	0.06
eastward wind (quadratic)	−4.00	−8.17	0.13	0.03
northward wind (linear)	−2.88	−7.36	1.55	0.10
northward wind (quadratic)	−1.83	−6.21	2.49	0.20
change in eastward wind	−0.34	−0.52	−0.16	0.00
change in northward wind	−0.16	−0.33	0.01	0.03
pressure change	0.13	−0.15	0.41	0.81
cloud cover	−0.20	−0.36	−0.05	0.01
precipitation (rain)	−0.74	−1.49	−0.06	0.02
species (Greater Whitethroat)	−0.02	−0.53	0.49	0.46
species (Greater Whitethroat) × pressure change	0.35	−0.06	0.75	0.95
species (Sedge Warbler)	−0.58	−1.11	−0.04	0.02
species (Sedge Warbler) × pressure change	0.05	−0.39	0.50	0.60

### 3.2. Routing

**Figure 4.**
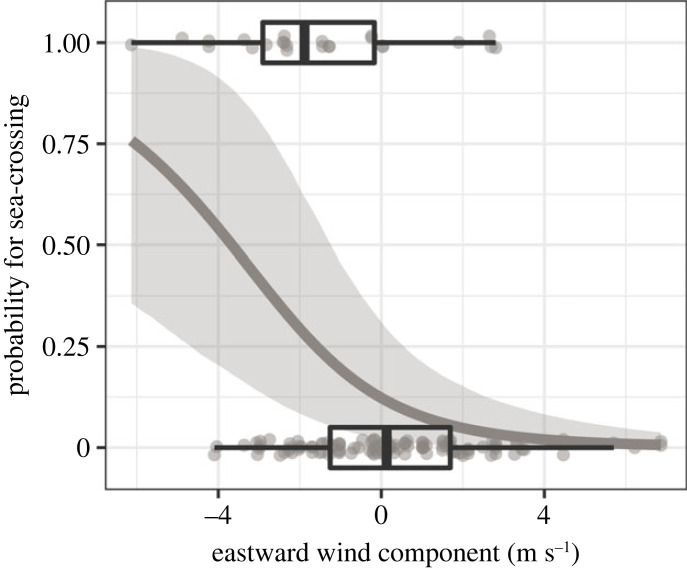
Influence of eastward (u) wind component on routing decision, predicted by the final binary logistic regression. Predictions (line) and 95% credible intervals (shaded area) are given together with the observed raw data (dots, boxplots). Predictions were made with the remaining numeric model covariates set to their means.

Fifteen Garden Warblers and nine Greater Whitethroats flew offshore and crossed the German Bight while all Sedge Warblers followed the coastline (table 1). Routing decisions differed between and within nights, with individuals taking different routes within the same night on eleven occasions. The probability for an offshore flight in both species was affected by the eastward (*u*) wind component, i.e. it increased with westward winds (table 3, figure 4). Within the night of departure, birds that took an offshore route departed on average half an hour earlier (1.4 ± 0.8 h after sunset; mean ± s.d.) than birds that followed the coastline (1.9 ± 1.2 h after sunset).

**Table 3.** Influence of eastward (u) wind component and species on routing decision, i.e. the probability that birds migrated offshore, in Garden Warblers and Greater Whitethroats. Estimates, 95% credible intervals and posterior probabilities for β > 0 are given for the final binary logistic regression.

parameter	estimate	2.5%	97.5%	*P*(β > 0)
intercept	−2.16	−3.86	−0.80	0.00
eastward wind	−1.40	−2.59	−0.59	0.00

### 3.3. Landing

**Figure 5.**
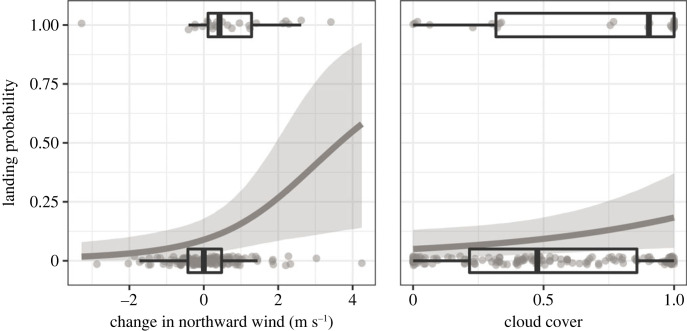
Influence of change in northward (v) wind and cloud cover on landing decision of individually tracked night-migratory songbirds during migratory endurance flight, predicted by the final binary logistic regression. Positive values of changing northward wind can be due to either freshening-up northward wind or a drop in southward wind. Predictions (lines) and 95% credible intervals (shaded areas) are given together with the observed raw data (dots, boxplots). Predictions were made with the remaining numeric model covariates set to their means.

We detected 24 landings within the study area after a sustained flight (table 1). Landing probability increased with positive changes in the northward (*v*) wind component. Such a change can be due to either freshening-up northward wind or a drop in southward wind. Additionally, landing probability increased with cloud cover (table 4, figure 5).

**Table 4.** Influence of change in northward (v) wind component and cloud cover on the landing probability in Garden Warblers, Greater Whitethroats and Sedge Warblers. Estimates, 95% credible intervals and posterior probabilities for β > 0 are given for the final binary logistic regression.

parameter	estimate	2.5%	97.5%	*P*(β > 0)
intercept	−2.32	−3.43	−1.45	0.00
change in northward wind	0.66	0.14	1.20	0.99
cloud cover	0.56	0.00	1.15	0.98

## 4. Discussion

Our study demonstrates that songbird migrants departed under favourable weather conditions (no precipitation and clear skies, also indicated by positive air pressure change), especially under supporting (westward and southward) wind directions from coastal stopover sites in Central Europe during autumn migration. Routing decisions, i.e. whether to fly offshore or to follow the coastline, are predicted by wind direction with westward winds increasing the probability for offshore flights. During a migratory endurance flight, more birds decided to land under overcast conditions or when the northward wind component increased, which is due to either freshening-up northward wind or a drop in (supporting) southward wind. Considering departure, routing and landing decisions in concert, we highlight the importance of weather as a common driver across all migratory decisions and studied species. Individual tracking between stopovers, and thus capturing the decision when to interrupt the migratory endurance flight revealed changes in wind support and cloud cover as potential triggers for a landing. These results support the hypothesis that avoidance of adverse weather conditions is an important ecological function of stopovers [3].

### 4.1. Departure

Birds migrating south or southwest in Central Europe during autumn predominantly experience unfavourable wind conditions, as the prevailing wind directions are west and southwest [61]. We found that both wind components predicted the departure probability from coastal stopover, indicating that songbirds select for light westward winds and preferred southward wind directions, i.e. tailwind, to depart. These findings match radar observations of more general migration patterns [37,62] and a telemetry study with thrushes [39] in our study region. Moreover, birds more likely departed when atmospheric pressure increased compared to the previous day. Atmospheric pressure and its change can serve as indicators to predict cloud, precipitation and turbulence conditions [35], and birds are assumed to use this as a cue to adjust their behaviour accordingly, with increasing air pressure often promoting departures [63,64]. These results are in line with previous studies [65–67] and fit our findings that departure probability positively correlated with cloud cover, suggesting a preference for clear skies. Moreover, birds select for periods with no precipitation to depart, since rain can be a severe hazard for small birds during flight [32].

In combination, certain weather parameters affect departure decisions in migratory songbirds and shape bird migration patterns [34,37]. Our data revealed departure peaks in six nights representing 40% of all detected flights. Such concentrations of nocturnal bird migration occurred in nights after phases with unfavourable weather conditions and were indicated by winds shifting to east and north, clear skies and no precipitation (electronic supplementary material, S4–S6). In other words, unfavourable weather conditions, especially headwinds, can lead to accumulations of birds ready to depart from stopover. Our data demonstrate how individual departure decisions cause this effect, also known as ‘Zugstau’ [35,68].

### 4.2. Routing

Once birds had departed from their initial stopover site, 24 (13.5%) migrated offshore and crossed the German Bight while the majority of 154 individuals followed the coastline. Since the spatial coverage of the receiver network is much higher along the coast compared to the open sea, it is, however, more likely to detect flights along the coast. Although most birds that cross the German Bight should arrive at the coast within the range of our receiver network [69], sea-crossings may have occurred undetected and the proportion of offshore flights should be considered as conservative, as their total number likely is underestimated.

Routing varied with the eastward wind component, which is consistent with the findings of Brust *et al*. [39] for short-distance migratory thrushes from the same study area. As birds had already selected favourable conditions to resume their migration, sufficient wind support seems to be the only weather-related basis of decision-making to fly offshore instead of following the coast. Moreover, birds that migrated offshore departed slightly earlier within the night of departure compared to birds that followed the coast [70]. By doing so, birds maximize the night time available for flying across the open sea and thereby minimize the exposure to the disadvantages of daytime flights, i.e. more turbulent air [18,71] and higher predation risk [72]. These findings highlight the importance of wind conditions in the context of barrier crossing [24,40,66], even of comparatively small ones such as the German Bight [39] or the Baltic Sea [73]. Although wind drift can cause substantial displacements in migratory birds [36], this passive effect is unlikely to be involved in this case, because (i) birds did not depart in strong winds, (ii) all Sedge Warblers were able to stay in close proximity to the coast, irrespective of the prevailing wind conditions and (iii) we simultaneously detected contrary routing decisions of different individuals. These individual differences within the same nights confirm that routing decisions are a particularly flexible and complex part of migration, which cannot be explained by extrinsic factors alone [30].

### 4.3. Landing

Even if birds departed from stopover under favourable conditions, they may experience a more or less rapid deterioration of weather conditions en route. Such conditions might ‘force’ birds to lower their flight altitude and search for stopover or ‘rescue’ sites (e.g. [33,44]). Especially under poor visibility due to rain or fog, birds might collide with anthropogenic obstacles such as tall buildings or wind turbines [74,75]. This is particularly problematic offshore, where songbirds find hardly any options to land and get attracted by illuminated artificial structures, which can lead to mass mortality of disoriented migrants [75–78]. Thus, knowledge about timing and routing is crucial to understand interactions of migrating birds with human activities and their potential consequences for individual survival and reproductive fitness [78,79].

Within our study area, we detected 24 landings after a sustained flight. However, due to the spatially restricted telemetry network, the proportion of landings in proximity of the study area is likely to be underestimated. Birds that experienced a positive change in the northward wind component during flight were more likely to suspend migration. In Central Europe, such a change in wind implies increasing headwind or at least a decrease in wind support for autumn migrants. While time-selected migrants, e.g. males during spring migration, may accept an increase in metabolic flight cost, birds that adopt other optimization criteria should show less risk-prone decisions and land rather than spend extra energy on flying under unfavourable conditions or even risk to perish at sea [3,33]. This especially applies to autumn migration, when birds favour a slower, less energetically costly migration compared to spring [8,10].

Stopovers fulfil multiple ecological functions like energy accumulation and physiological recovery [3]. It is furthermore assumed that stopovers serve to avoid adverse environmental conditions for migratory endurance flights (e.g. [3,27]). However, what actually initiates stopovers, i.e. the landing decision, remains fairly unstudied (but see [33]). Using individual tracking between stopovers, we here show that the avoidance presumption is reasonably compatible with our songbird data. Effectively, our results can help to better understand the functions of stopover, e.g. minimizing the costs of transport, and its ecological role in the annual cycle.

## 5. Conclusion

Departure, routing and landing decisions of long-distance migrants at the edge of an ecological barrier highly depend on weather conditions, primarily wind and precipitation. These weather parameters inherently show spatio-temporal changes and concentrate bird migration to phases with conditions favourable for migration. However, adjustment of migration behaviour en route to local conditions is highly variable between and within individuals.

Our data extend the understanding of weather as a common driver for migration and stopover behaviour across all migratory decisions. These findings may also be transferrable to other taxa and regions. Based on individual tracking between stopovers, we identify deteriorating weather conditions as a potential proximate landing cue, supposing avoidance of adverse weather conditions as an important function of stopover [3].

## Data Availability

The data are provided in the electronic supplementary material [80].

## References

[1] Hahn S, Bauer S, Liechti F. 2009 The natural link between Europe and Africa—2.1 billion birds on migration. Oikos **118**, 624-626. (10.1111/j.1600-0706.2008.17309.x)

[2] Briedis M et al. 2020 Broad-scale patterns of the Afro-Palaearctic landbird migration. Glob. Ecol. Biogeogr. **29**, 722-735. (10.1111/geb.13063)

[3] Schmaljohann H, Eikenaar C, Sapir N. 2022 Understanding the ecological and evolutionary function of stopover in migrating birds. Biol. Rev. **97**, 1231-1252. (10.1111/brv.12839) 35137518

[4] Bruderer B, Liechti F. 1998 Flight behaviour of nocturnally migrating birds in coastal areas: crossing or coasting. J. Avian Biol. **29**, 499-507. (10.2307/3677169)

[5] Alerstam T, Lindström Å. 1990 Optimal bird migration: the relative importance of time, energy, and safety. In Bird migration (ed. E Gwinner), pp. 331-351. Berlin, Germany: Springer.

[6] Green M, Alerstam T, Clausen P, Drent R, Ebbinge BS. 2002 Dark-bellied Brent geese *Branta bernicla bernicla*, as recorded by satellite telemetry, do not minimize flight distance during spring migration. Ibis **144**, 106-121. (10.1046/j.0019-1019.2001.00017.x)

[7] Schmaljohann H, Fox JW, Bairlein F. 2012 Phenotypic response to environmental cues, orientation and migration costs in songbirds flying halfway around the world. Anim. Behav. **84**, 623-640. (10.1016/j.anbehav.2012.06.018)

[8] Schmaljohann H. 2018 Proximate mechanisms affecting seasonal differences in migration speed of avian species. Sci. Rep. **8**, 4106. (10.1038/s41598-018-22421-7) 29515154 PMC5841396

[9] Schmaljohann H, Both C. 2017 The limits of modifying migration speed to adjust to climate change. Nat. Clim. Chang. **7**, 573-576. (10.1038/nclimate3336)

[10] Nilsson C, Klaassen RH, Alerstam T. 2013 Differences in speed and duration of bird migration between spring and autumn. Am. Nat. **181**, 837-845. (10.1086/670335) 23669545

[11] Alerstam T. 2001 Detours in bird migration. J. Theoret. Biol. **209**, 319-331. (10.1006/jtbi.2001.2266) 11312592

[12] Briedis M et al. 2019 A full annual perspective on sex-biased migration timing in long-distance migratory birds. Proc. R. Soc. B **286**, 20182821. (10.1098/rspb.2018.2821) PMC6408886 30963841

[13] Mitchell GW, Woodworth BK, Taylor PD, Norris DR. 2015 Automated telemetry reveals age specific differences in flight duration and speed are driven by wind conditions in a migratory songbird. Mov. Ecol. **3**, 19. (10.1186/s40462-015-0046-5) 26279850 PMC4537592

[14] Alerstam T. 2011 Optimal bird migration revisited. J. Ornithol. **152**, 5-23. (10.1007/s10336-011-0694-1)

[15] Dorka V. 1966 Das jahres-und tageszeitliche Zugmuster von Kurz- und Langstreckenziehern nach Beobachtungen auf den Alpenpässen Cou Bretolet (Wallis). Ornithologischer Beobachter **63**, 165-223.

[16] Alerstam T. 2009 Flight by night or day? Optimal daily timing of bird migration. J. Theoret. Biol. **258**, 530-536. (10.1016/j.jtbi.2009.01.020) 19459237

[17] Delingat J, Dierschke V, Schmaljohann H, Mendel B, Bairlein F. 2006 Daily stopovers as optimal migration strategy in a long-distance migrating passerine: the northern wheatear *Oenanthe oenanthe*. Ardea **94**, 593-605.

[18] Müller F, Taylor PD, Sjöberg S, Muheim R, Tsvey A, Mackenzie SA, Schmaljohann H. 2016 Towards a conceptual framework for explaining variation in nocturnal departure time of songbird migrants. Mov. Ecol. **4**, 24. (10.1186/s40462-016-0089-2) 27833750 PMC5066284

[19] Gwinner E. 1996 Circadian and circannual programmes in avian migration. J. Exp. Biol. **199**, 39-48. (10.1242/jeb.199.1.39) 9317295

[20] Berthold P. 1973 Relationships between migratory restlessness and migration distance in six Sylvia species. Ibis **115**, 594-599. (10.1111/j.1474-919X.1973.tb01998.x)

[21] Schmaljohann H, Lisovski S, Bairlein F. 2017 Flexible reaction norms to environmental variables along the migration route and the significance of stopover duration for total speed of migration in a songbird migrant. Front. Zool. **14**, 17. (10.1186/s12983-017-0203-3) 28344630 PMC5360013

[22] Goymann W, Spina F, Ferri A, Fusani L. 2010 Body fat influences departure from stopover sites in migratory birds: evidence from whole-island telemetry. Biol. Lett. **6**, 478-481. (10.1098/rsbl.2009.1028) 20164077 PMC2936206

[23] Schmaljohann H, Korner-Nievergelt F, Naef-Daenzer B, Nagel R, Maggini I, Bulte M, Bairlein F. 2013 Stopover optimization in a long-distance migrant: the role of fuel load and nocturnal take-off time in Alaskan northern wheatears (*Oenanthe oenanthe*). Front. Zool. **10**, 26. (10.1186/1742-9994-10-26) 23663358 PMC3665591

[24] Deppe JL et al. 2015 Fat, weather, and date affect migratory songbirds’ departure decisions, routes, and time it takes to cross the Gulf of Mexico. Proc. Natl Acad. Sci. USA **112**, E6331-E6338. (10.1073/pnas.1503381112) 26578793 PMC4655507

[25] Hegemann A, Abril PA, Muheim R, Sjöberg S, Alerstam T, Nilsson JÅ, Hasselquist D. 2018 Immune function and blood parasite infections impact stopover ecology in passerine birds. Oecologia **188**, 1011-1024. (10.1007/s00442-018-4291-3) 30386941 PMC6244813

[26] Brust V, Eikenaar C, Packmor F, Schmaljohann H, Hüppop O, Czirják GÁ. 2022 Do departure and flight route decisions correlate with immune parameters in migratory songbirds? Funct. Ecol. **36**, 3007-3021. (10.1111/1365-2435.14187)

[27] Carneiro C, Gunnarsson TG, Alves JA. 2020 Linking weather and phenology to stopover dynamics of a long-distance migrant. Front. Ecol. Evol. **8**, 145. (10.3389/fevo.2020.00145)

[28] Hewson CM, Thorup K, Pearce-Higgins JW, Atkinson PW. 2016 Population decline is linked to migration route in the common cuckoo. Nat. Commun. **7**, 12296. (10.1038/ncomms12296) 27433888 PMC4960304

[29] Ward MP, Benson TJ, Deppe J, Zenzal Jr TJ, Diehl RH, Celis-Murillo A, Bolus R, Moore FR. 2018 Estimating apparent survival of songbirds crossing the Gulf of Mexico during autumn migration. Proc. R. Soc. B **285**, 20181747. (10.1098/rspb.2018.1747) PMC6234880 30355710

[30] Stanley CQ, MacPherson M, Fraser KC, McKinnon EA, Stutchbury BJ. 2012 Repeat tracking of individual songbirds reveals consistent migration timing but flexibility in route. PLoS ONE **7**, e40688. (10.1371/journal.pone.0040688) 22848395 PMC3405083

[31] Åkesson S, Helm B. 2020 Endogenous programs and flexibility in bird migration. Front. Ecol. Evol. **8**, 78. (10.3389/fevo.2020.00078)

[32] Newton I. 2007 Weather-related mass-mortality events in migrants. Ibis **149**, 453-467. (10.1111/j.1474-919X.2007.00704.x)

[33] Kelsey NA, Hüppop O, Bairlein F. 2021 Days to visit an offshore island: effect of weather conditions on arrival fuel load and potential flight range for common blackbirds *Turdus merula* migrating over the North Sea. Mov. Ecol. **9**, 53. (10.1186/s40462-021-00290-6) 34674773 PMC8529821

[34] Erni B, Liechti F, Underhill LG, Bruderer B. 2002 Wind and rain govern the intensity of nocturnal bird migration in central Europe—a log-linear regression analysis. Ardea **90**, 155-166.

[35] Richardson W. 1990 Timing of bird migration in relation to weather: updated review. In Bird migration (ed. E Gwinner), pp. 78-101. Berlin, Germany: Springer.

[36] Liechti F. 2006 Birds: blowin’ by the wind? J. Ornithol. **147**, 202-211. (10.1007/s10336-006-0061-9)

[37] Nilsson C et al. 2019 Revealing patterns of nocturnal migration using the European weather radar network. Ecography **42**, 876-886. (10.1111/ecog.04003)

[38] Packmor F, Klinner T, Woodworth BK, Eikenaar C, Schmaljohann H. 2020 Stopover departure decisions in songbirds: do long-distance migrants depart earlier and more independently of weather conditions than medium-distance migrants? Mov. Ecol. **8**, 6. (10.1186/s40462-020-0193-1) 32047634 PMC7006082

[39] Brust V, Michalik B, Hüppop O. 2019 To cross or not to cross—thrushes at the German North Sea coast adapt flight and routing to wind conditions in autumn. Mov. Ecol. **7**, 32. (10.1186/s40462-019-0173-5) 31695918 PMC6824093

[40] Dossman BC, Mitchell GW, Norris DR, Taylor PD, Guglielmo CG, Matthews SN, Rodewald PG. 2016 The effects of wind and fuel stores on stopover departure behavior across a migratory barrier. Behav. Ecol. **27**, 567-574. (10.1093/beheco/arv189)

[41] Bairlein F, Dierschke J, Dierschke V, Salewski V, Geiter O, Hüppop K, Köppen U, Fiedler W. 2014 Atlas des Vogelzugs. Ringfunde deutscher Brut-und Gastvögel. Wiebelsheim, Germany: Aula-Verlag.

[42] Taylor P et al. 2017 The Motus Wildlife Tracking System: a collaborative research network to enhance the understanding of wildlife movement. Avian Conserv. Ecol. **12**, 120108. (10.5751/ACE-00953-120108)

[43] Schaub M, Liechti F, Jenni L. 2004 Departure of migrating European robins, *Erithacus rubecula*, from a stopover site in relation to wind and rain. Anim. Behav. **67**, 229-237. (10.1016/j.anbehav.2003.03.011)

[44] Dierschke J, Dierschke V, Hüppop K, Hüppop O, Jachmann KF. 2011 Die Vogelwelt der Insel Helgoland. Helgoland, Germany: OAG Helgoland.

[45] Naef-Daenzer B. 2007 An allometric function to fit leg-loop harnesses to terrestrial birds. J. Avian Biol. **38**, 404-407. (10.1111/j.2007.0908-8857.03863.x)

[46] Schmaljohann H, Naef-Daenzer B. 2011 Body condition and wind support initiate the shift of migratory direction and timing of nocturnal departure in a songbird. J. Anim. Ecol. **80**, 1115-1122. (10.1111/j.1365-2656.2011.01867.x) 21615404

[47] Brzustowski J, LePage D. 2021 Motus: fetch and use data from the Motus Wildlife Tracking System. R package version 4.0.6.

[48] Schaub M, Jenni L. 2001 Stopover durations of three warbler species along their autumn migration route. Oecologia **128**, 217-227. (10.1007/s004420100654) 28547471

[49] Smetzer JR, King DI, Taylor PD. 2017 Fall migratory departure decisions and routes of blackpoll warblers *Setophaga striata* and red-eyed vireos *Vireo olivaceus* at a coastal barrier in the Gulf of Maine. J. Avian Biol. **48**, 1451-1461. (10.1111/jav.01450)

[50] Michalik B, Brust V, Hüppop O. 2020 Are movements of daytime and nighttime passerine migrants as different as day and night? Ecol. Evol. **10**, 11 031-11 042. (10.1002/ece3.6704) 33144946 PMC7593151

[51] Hersbach H et al. 2018 ERA5 hourly data on single levels from 1979 to present. Copernicus Climate Change Service (C3S) Climate Data Store (CDS) (accessed 12 January 2022). (10.24381/cds.adbb2d47)

[52] Muñoz-Sabater J et al. 2021 ERA5-Land: a state-of-the-art global reanalysis dataset for land applications. Earth Syst. Sci. Data **13**, 4349-4383. (10.5194/essd-13-4349-2021)

[53] Bruderer B, Peter D, Korner-Nievergelt F. 2018 Vertical distribution of bird migration between the Baltic Sea and the Sahara. J. Ornithol. **159**, 315-336. (10.1007/s10336-017-1506-z)

[54] DWD Climate Data Center (CDC). 2022 Recent hourly station observations of precipitation for Germany, quality control not completed yet, version recent. See https://opendata.dwd.de/climate_environment/CDC/observations_germany/climate/hourly/precipitation/recent/ (accessed 14 February 2022).

[55] DWD Climate Data Center (CDC). 2021 Historical hourly station observations of precipitation for Germany. Version v21.3. See https://opendata.dwd.de/climate_environment/CDC/observations_germany/climate/hourly/precipitation/historical/ (accessed 14 February 2022).

[56] Brilleman SL, Elci EM, Novik JB, Wolfe R. 2020 Bayesian survival analysis using the rstanarm R package. (https://arxiv.org/abs/2002.09633)

[57] Goodrich B, Gabry J, Ali I, Brilleman S. 2020 Rstanarm: Bayesian applied regression modeling via stan. R package version 2.21.2.

[58] R Core Team. 2021 R: a language and environment for statistical computing. Vienna, Austria: R Foundation for Statistical Computing.

[59] Bürkner PC. 2017 brms: an R package for Bayesian multilevel models using Stan. J. Stat. Softw. **80**, 1-28. (10.18637/jss.v080.i01)

[60] Vehtari A, Gelman A, Gabry J. 2017 Practical Bayesian model evaluation using leave-one-out cross-validation and WAIC. Stat. Comput. **27**, 1413-1432. (10.1007/s11222-016-9696-4)

[61] Kemp MU, Shamoun-Baranes J, Van Gasteren H, Bouten W, Van Loon EE. 2010 Can wind help explain seasonal differences in avian migration speed? J. Avian Biol. **41**, 672-677. (10.1111/j.1600-048X.2010.05053.x)

[62] Bradarić M, Bouten W, Fijn RC, Krijgsveld KL, Shamoun-Baranes J. 2020 Winds at departure shape seasonal patterns of nocturnal bird migration over the North Sea. J. Avian Biol. **51**, e02562. (10.1111/jav.02562)

[63] Åkesson S, Walinder G, Karlsson L, Ehnbom S. 2002 Nocturnal migratory flight initiation in reed warblers *Acrocephalus scirpaceus*: effect of wind on orientation and timing of migration. J. Avian Biol. **33**, 349-357. (10.1034/j.1600-048X.2002.02951.x)

[64] Metcalfe J, Schmidt KL, Kerr WB, Guglielmo CG, MacDougall-Shackleton SA. 2013 White-throated sparrows adjust behaviour in response to manipulations of barometric pressure and temperature. Anim. Behav. **86**, 1285-1290. (10.1016/j.anbehav.2013.09.033)

[65] Åkesson S, Walinder G, Karlsson L, Ehnbom S. 2001 Reed warbler orientation: initiation of nocturnal migratory flights in relation to visibility of celestial cues at dusk. Anim. Behav. **61**, 181-189. (10.1006/anbe.2000.1562) 11170708

[66] Woodworth BK, Mitchell GW, Norris DR, Francis CM, Taylor PD. 2015 Patterns and correlates of songbird movements at an ecological barrier during autumn migration assessed using landscape- and regional-scale automated radiotelemetry. Ibis **157**, 326-339. (10.1111/ibi.12228)

[67] Dänhardt J, Lindström Å. 2001 Optimal departure decisions of songbirds from an experimental stopover site and the significance of weather. Anim. Behav. **62**, 235-243. (10.1006/anbe.2001.1749)

[68] Schüz E. 1952 Vom Vogelzug: Grundriss der Vogelzugskunde. Frankfurt, Germany: Paul Schöps.

[69] Brust V, Hüppop O. 2021 Underestimated scale of songbird offshore migration across the south-eastern North Sea during autumn. J. Ornithol. **163**, 51-60. (10.1007/s10336-021-01934-5)

[70] Müller F, Eikenaar C, Crysler ZJ, Taylor PD, Schmaljohann H. 2018 Nocturnal departure timing in songbirds facing distinct migratory challenges. J. Anim. Ecol. **87**, 1102-1115. (10.1111/1365-2656.12821) 29504627

[71] Kerlinger P, Moore FR. 1989 Atmospheric structure and avian migration. In Current ornithology (ed. DM Power), pp. 109-142. Berlin, Germany: Springer.

[72] Walter H. 1979 Eleonora's falcon: adaptations to prey and habitat in a social raptor. Chicago, IL: University of Chicago Press.

[73] Sjöberg S, Alerstam T, Åkesson S, Schulz A, Weidauer A, Coppack T, Muheim R. 2015 Weather and fuel reserves determine departure and flight decisions in passerines migrating across the Baltic Sea. Anim. Behav. **104**, 59-68. (10.1016/j.anbehav.2015.02.015)

[74] Marques AT, Batalha H, Rodrigues S, Costa H, Pereira MJR, Fonseca C, Mascarenhas M, Bernardino J. 2014 Understanding bird collisions at wind farms: an updated review on the causes and possible mitigation strategies. Biol. Conserv. **179**, 40-52. (10.1016/j.biocon.2014.08.017)

[75] Hüppop O, Hüppop K, Dierschke J, Hill R. 2016 Bird collisions at an offshore platform in the North Sea. Bird Study **63**, 73-82. (10.1080/00063657.2015.1134440)

[76] Shamoun-Baranes J, van Gasteren H. 2011 Atmospheric conditions facilitate mass migration events across the North Sea. Anim. Behav. **81**, 691-704. (10.1016/j.anbehav.2011.01.003)

[77] Dierschke V, Rebke M, Hill K, Weiner CN, Aumüller R, Hill R. 2021 Auswirkungen der Beleuchtung maritimer Bauwerke auf den nächtlichen Vogelzug über dem Meer. Natur und Landschaft **96**, 282-292. (10.17433/6.2021.50153915.282-292)

[78] Hüppop O, Michalik B, Bach L, Hill R, Pelletier SK. 2019 Migratory birds and bats. In Wildlife and windfarms, conflicts and solutions. Volume 3 offshore: potential effects (ed. MR Perrow), pp. 142-173. Exeter, UK: Pelagic publishing.

[79] Gauld JG et al. 2022 Hotspots in the grid: avian sensitivity and vulnerability to collision risk from energy infrastructure interactions in Europe and North Africa. J. Appl. Ecol. **59**, 1496-1512. (10.1111/1365-2664.14160)

[80] Rüppel G, Hüppop O, Lagerveld S, Schmaljohann H, Brust V. 2023 Departure, routing and landing decisions of long-distance migratory songbirds in relation to weather. *Figshare*. (10.6084/m9.figshare.c.6403996) PMC9905979 36778957

